# Suicidality in Women with Adjustment Disorder and Depressive Episodes Attending an Irish Perinatal Mental Health Service

**DOI:** 10.3390/ijerph16203970

**Published:** 2019-10-18

**Authors:** Anne M Doherty, Genevieve Crudden, Faraz Jabbar, John D Sheehan, Patricia Casey

**Affiliations:** 1 Department of Psychiatry, University Hospital Galway, Galway H91 YR71, Ireland; Cruddeng@tcd.ie; 2 School of Medicine, National University of Ireland Galway, Galway H91 TK33, Ireland; 3 Department of Psychiatry, University Hospital of Northern British Columbia, 1444 Edmonton Street, Prince George, BC V2M6W5, Canada; farazjabbar@hotmail.com; 4 Rotunda Hospital, Parnell Square, Dublin 1 DO1 P5W9, Ireland; sheehanj@mater.ie; 5 Mater University Hospital, 63 Eccles Street, Dublin 7 D07 R2WY, Ireland; profcasey@esatclear.ie

**Keywords:** perinatal care, adjustment disorder, depressive episode, postpartum depression, psychiatry

## Abstract

Depression is common in the perinatal period, with prevalence rates of 14.4%, but prevalence rates of adjustment disorder in this period have not been established. We aimed to examine the characteristics of women attending a perinatal psychiatry service diagnosed with adjustment disorder (AD) or depressive episodes (DE). The data were collected as part of a multicentre case-control study of 370 patients, 45 of whom were recruited from perinatal psychiatry service at a maternity hospital. We recruited 45 patients with AD or DE diagnosed in the perinatal period and compared them to a matched sample of 109 non-perinatal women. Almost half, 22 (48.9%) perinatal women had a diagnosis of AD and 23 (51.1%) had a diagnosis of DE. Of the perinatal participants, those with AD had more stressful life events, and suicidal ideation and behaviours were three times more common (31.8%) in AD than in DE (8.7%). There were no significant differences in levels of suicidality between the perinatal and the non-perinatal groups. In our cohort, AD is associated with symptoms of depression including suicidal ideation during the perinatal period. Further study is required to examine the relationship between stressors and suicidality in this population.

## 1. Introduction

Depressive illnesses are common in the perinatal period (pregnancy and the first postnatal year), especially adjustment disorder (AD) and depressive episodes (DE). The postpartum period is associated with higher rates of AD, generalised anxiety disorder and DE [1]. The only Irish study to examine perinatal depression reported community prevalence rates of 14% [2], which are consistent with international prevalence rates of 10%–15% [3].

DE is a disorder characterised by a number of symptoms and associated impairment of functioning. The two international diagnostic classification systems require the presence of 3 core symptoms (low mood, reduced energy and reduced interest) for a diagnosis to be made, along with a number of other biological and cognitive symptoms [4,5].

AD is a condition arising within three months of a known stressor and resolving spontaneously when the stressor is removed or with the passage of time [4,5]. There is overlap in symptoms with depressive episodes and anxiety disorders. The context is important in making this diagnosis: A triggering stressor is required. A previous study reported that while AD may be perceived as a subthreshold condition, it is associated with depressive symptoms in the moderate range and with equivalent levels of suicidality to DE [6].

Data from the UK suggest that pregnancy itself is not associated with an increased risk of a new psychiatric diagnosis, but there is an increased risk of recurrence or deterioration of pre-existing diagnoses. Data from the US which used a semistructured diagnostic tool reported that pregnancy is not associated with an increased risk of mental illness and reported an increased incidence of mental disorder in the postpartum period [7].

Gelaye et al. reported a higher level of suicidal ideation in pregnant women compared with the general population [8]. This systematic review identified predictors of suicidal ideation in perinatal women, including depression, intimate partner violence and educational disadvantage, and called for increased screening of this at-risk population. It did not comment on AD in this population, likely because it is an under-researched condition and not examined in the literature included. Of note, this study attempted to explain ante-partum suicidal ideation using a ‘stress-diathesis model’, which may be relevant to the women with stress-related conditions, such as AD [8]. As suicide is a leading cause of maternal mortality in the developed world, responsible for 28% of maternal deaths in the UK, depressive illnesses are therefore a modifiable risk factor for perinatal mortality [9,10]. Addressing perinatal depressive disorders has been identified as a maternal health priority by the WHO, as a Millennium Development Goal in 2008 [11]. 

Although many studies have screened for depression in the perinatal period, there is a dearth of more research which utilises more robust design examining clinical diagnosis or structured diagnostic tools. In the area of stress-related mental disorders, while there have been a number of heterogeneous studies examining perinatal post-traumatic stress disorder, no previous studies have examined the role of stress-disorders, namely, AD in the perinatal period [12]. 

We aimed to examine the presenting symptoms of women attending the perinatal psychiatry service at a Dublin maternity hospital, who were diagnosed with AD or DE, and to compare them to a comparison group recruited from the liaison psychiatry services at two other Dublin hospitals (from the emergency department, medical wards and outpatient clinics) matched for age, gender, and clinical diagnosis. Our clinical experience seemed to indicate that AD in the perinatal period in women was associated with suicidal ideations and behaviours and is, therefore, a serious diagnosis that merits clinical attention. Our study was designed to explore this further.

## 2. Materials and Methods

This is a post hoc analysis of a multicentre study of AD and DE in three Dublin hospitals, one of which is a maternity hospital. The methodology of this study has previously been described in detail in other published papers [6,13]. We compared those participants recruited from the perinatal psychiatry service at one of Ireland’s three National Maternity Hospitals, who were diagnosed by the perinatal psychiatrists with either DE or AD, based on the ICD-10 diagnostic criteria, with a matched sample from the other two general medical hospitals. The exclusion criteria were as follows: primary diagnosis of a substance misuse disorder, cognitive impairment, incapable of giving informed consent, under 18, psychotic symptoms or not fluent in the English language.

Patients were interviewed by a researcher blind to the clinical diagnosis using validated instruments. The validated instruments used included:

1. SCAN—Schedules for Clinical Assessment in Neuropsychiatry, a clinician-administered structured interview schedule providing diagnoses based on ICD-10; its limitations include the categorisation of AD in a section entitled “Inferences and Attributions”, only used if caseness for another diagnosis has not been reached [14].
2. IDS-C30—Inventory of Depressive Symptoms—Clinician Rated is a 30-item clinician administered schedule of depressive symptoms. In this paper, question 18 was used to measure suicidal ideation and behaviour relevant to this study [15].
3. The List of Threatening Experiences was used to assess significant life events (preceding 6 months). It comprises 12 questions, for 12 different traumatic life events (range 0–12) [16].
4. SAPAS Standardised Assessment of Personality—Abbreviated Scale is an 8-item self-rated screen for personality disorder, where a score of three or more indicates a probable personality disorder [17].
5. Oslo Social Support Scale is a self-rated instrument which assesses perception of social support (range 3–14): A higher score indicates greater perceived support [18].
6. SIS—Suicide Intent Scale is a 15-item self-report questionnaire which measures severity of intent in patients presenting with suicidal behaviour [19].
7. SSI—Scale of Suicidal Ideation is a 19-item self-report schedule assessing suicidal ideation in individuals who did not present following suicidal behaviour [20].

We used three different measures to assess suicidality:

- All participants completed the IDS-C30. Question 18 (Q18), which evaluates suicidality across the spectrum of nil, passive death wishes, suicidal ideation and suicidal behaviour [15].
- Participants who presented following an act of suicidal behaviour completed the SIS.
- Participants who did not present with suicidal behaviour completed the SSI which measured the severity of suicidal ideation.

Structured interviews for AD are poorly developed: Mostly where AD is included, the diagnosis is only made if the threshold for another disorder is not met. This approach ignores context and has attracted criticism from researchers of stress-related disorders [21,22]. Therefore, although we used SCAN, a structured interview, for the purposes of this study, we used clinical diagnosis as the diagnostic gold standard.

We compared women during the perinatal period recruited from a maternity hospital, with a sample matched for age, gender and diagnosis recruited from the other two hospitals in this study. This was conducted by including all patients recruited to this study (all with clinical diagnoses of AD or DE) from the perinatal psychiatry services and matching with all female patients recruited from the liaison psychiatry services at the two other hospitals (emergency department, medical wards and outpatient settings) within the same age-range as the perinatal group (18–48 years). Given that this was a post hoc analysis and not adequately powered for this subgroup, for the comparison group, we included all participants in this analysis who met the criteria of age, gender and diagnosis, rather than choosing equal sample sizes. In the original study, 370 people were recruited, with 45 of these presented in the perinatal period, and we identified 109 matched controls from the female non-perinatal population recruited. 

Statistical analysis was conducted using SPSS (v20, SPSS Inc., Chicago, IL, USA). Bivariate analysis employed the use of independent samples *t*-test, and chi-square test to examine the differences in mean scores for demographic and clinical variables. Multivariate analysis was conducted using logistic regression, with perinatal status as the dependent variable.

## 3. Results

Of the forty-five perinatal women recruited to this study: 22 (48.9%) had a clinical diagnosis of AD and 23 (51.1%) were diagnosed with DE. The mean age was 34 years. A greater proportion of those with a diagnosis of DE were married (87%) than those with a diagnosis of AD (64%), although this difference was not statistically significant (Table 1).

We identified 109 female patients outside the perinatal period, aged 18–48, and the sociodemographic and clinical characteristics of the comparison group are described in Table 2. 

The perinatal population had significant depressive symptoms, with a mean IDSC-30 score of 37.1 (low severe range >36), without significant difference in the mean scores in AD and DE. Nearly two-thirds (64%) of this cohort screened positive for likely personality disorder, and although there were no significant differences between the two diagnostic groups, a higher proportion of women with a diagnosis of DE (70%) screened positive than with AD (59.1%). 

Across AD and DE in the perinatal group, 22.2% (*n* = 10) reported suicidal ideations or behaviours: This was more common in those with a diagnosis of AD (27.3%; *n* = 6) than of DE (17.3%; *n* = 4), although it did not reach statistical significance (*p* = 0.693). Three participants (6.7%) presented with suicidal behaviours, two of whom had a diagnosis of AD and one had a diagnosis of DE. The participants with AD had higher levels of intent as measured by SIS (nonstatistically significant). Of those who did not present with suicidal behaviours and completed the SSI, there were overall low scores, and the difference between mean scores in AD (mean 3.1; SD 5.3) and DE (mean 3.5; SD 5.9) was neither statistically significant nor clinically meaningful (Table 1).

There were no significant differences between the two diagnoses in the perinatal group in measures of social support or social functioning. Participants with AD reported twice the mean numbers of stressful life events compared with DE (1.0 event, SD1.3) as measured by the LTE (*p* = 0.026). When we examined specific life events, there were more life events in every category in the AD group compared to the DE group, except for two categories: marital separation and unemployment. None of the differences in any of the individual items between the two groups were statistically significant.

There were no statistically significant differences in depressive symptoms between the perinatal and non-perinatal groups in terms of depressive symptoms (*p* = 0.605), suicidality (*p* = 0.159), social support (*p* = 0.51) or personality disorder (*p* = 0.463), although there were trend greater proportions of patients with suicidal behaviours in the non-perinatal (21.3%) compared with the perinatal groups (6.7%). There were significantly less negative life events reported by those in the perinatal group (1.5 v 1.9; *p* = 0.012). The individuals in the non-perinatal group were significantly more likely to be unmarried.

We performed logistic regression, with perinatal status as the dependent variable. We did not find that any of the variables studied were appreciably different in the two groups. The levels of depressive symptoms and of suicidal ideation were not significantly lower in the perinatal group compared with the non-perinatal group.

## 4. Discussion

Women referred for psychiatric assessments in the perinatal period who were diagnosed with DE or AD presented with significant depressive symptoms of moderate to severe severity: There were no statistically significant differences in severity between those with a diagnosis of AD and of DE. One fifth reported suicidal ideation or behaviours, and a significant proportion presented with suicidal acts, which were less common in the perinatal group, although this did not reach statistical significance. Both suicidal ideation and behaviour were more common in those women with a clinical diagnosis of AD than DE.

There were no significant differences in depressive symptoms or suicidal ideation or behaviour between perinatal and non-perinatal patients on univariate or multivariate analysis. However, there were trends towards more suicidal ideation in the non-perinatal group. 

A previous study reported that although AD is often perceived as a subthreshold condition, it is associated with depressive symptoms in the moderate range and with equivalent levels of suicidality to DE [6]. This study is at variance with the findings of Gelaye et al., a systematic review which reported a higher level of suicidal ideation in pregnant women compared with the general population, although the differences in our population were not statistically significant [8]. Up to one third of maternal deaths are associated with suicide [9,23,24].

This is one of two Irish studies reported in the peer-reviewed literature examining DE in women with perinatal mental health disorders, and the only study which examines AD. Crotty and Sheehan reported prevalence rates of depression of 14.4% in their study population in Dublin [2]. 

The limitations of this study include the small numbers arising from the post hoc analysis, resulting in it being underpowered for multivariate analysis. However, as there is little published research on perinatal mental health problems in Ireland, and on perinatal AD internationally using semistructured interview schedules, this study is the largest to date.

Another potential limitation is the use of clinical diagnosis to diagnose AD. We utilised clinical diagnosis as the “gold standard” for this study as recommended by experts in this area [21]. Diagnosis in adjustment disorder (AD) has been the subject of much academic debate. Structured diagnostic tools such as SCAN use symptoms alone in arriving at a diagnosis without considering context, whereas context is key in arriving at a clinical diagnosis. As a result, there is a body of opinion that clinical diagnosis is the gold standard method for the diagnosis of AD [21,22], as clinical diagnosis, unlike structured interviews, considers both symptoms and their context [22,25,26,27]. Therefore, the use of clinical diagnosis may be regarded as either a strength or a limitation.

Although AD is often considered a subclinical disorder, this study suggests that it is associated with considerable depressive symptomatology in perinatal patients and has higher rates of suicidal ideation and behaviour than in DE. There were no significant differences in the rates of depression and suicidal ideation between the perinatal and non-perinatal groups, which supports previous evidence that this period may not be not protective against mental disorder or suicidal ideation and behaviours. AD may potentially represent an additional focus in addressing maternal mortality. 

## 5. Conclusions

An important diagnosis in the perinatal period, AD is associated with serious symptoms, including suicidal ideation and behaviours. Suicidal ideations and acts are common in pregnant women with AD and DE and are surprisingly more common in AD than in DE (not statistically significant), as these are stressful life events. A diagnosis of AD may be a serious one carrying risk of suicidal ideation and behaviours and should be considered as seriously as a diagnosis of DE, especially among perinatal patients, who require careful monitoring and supportive treatment. Further study is required to examine the relationship between stressors, AD and suicidal ideation in this population.

## Figures and Tables

**Table 1:** Demographic and clinical characteristics in the perinatal population—women recruited in the perinatal period (pregnancy to 12 months following birth).

Characteristics		Total *N* = 45	Adjustment Disorder * *N* = 22 (48.9%)	Depressive Episode * *N* = 23 (51.1%)	Statistic *p*-Value
Mean Age, years (SD)		34.0 (6.1)	33.8 (6.0)	34·2 (6.2)	0·809^a^
Marital Status	Single, *n* (%) Married/Cohabit, *n* (%) Other, *n* (%)	8 (18.1)	6 (27.3)	2 (8.7)	0·185 ^b^
		34 (75.4)	14 (63.6)	20 (87)	
		3 (6.5)	2 (9.1)	1 (4.3)	
Diagnosis on semi-structured interview (SCAN) AD (%) DE (%)		8 (17.8)	6 (27.3)	2 (8.7)	0.103 ^b^
		37 (82.2)	16 (72.7)	21 (91.3)	
Depressive symptoms: Mean IDSC-30 total score, range 0-90 (SD)		37.1 (14.9)	37.1 (14.7)	37·0 (15·4)	0·984 ^a^
Personality: Mean SAPAS score, range 0–8 (SD)		3.2 (1.7)	2·9 (1·6)	3·5 (1·8)	0·183 ^a^
Proportion of SAPAS score ≥ 3 (%)		29 (64.4)	13 (59.1)	16 (70)	0·259 ^a^
Social Support: Mean Oslo Social Support Scale score, range 3–14 (SD)		10.3 (2.4)	10·9 (2·5)	9·8 (2·2)	0·15 ^a^
**Life Events: Mean score on List of Threatening Events, Range 0–12 (SD)**		**1.5 (1.5)**	**2·1 (1·5)**	**1.0 (1.4)**	**0·026 ^a^**
Suicidality^c^	Nil Passive death wish Suicidal ideation Suicidal behaviour	26 (57.8)	13 (59.1)	13 (56.5)	0.693 ^b^
		9 (20)	3 (13.6)	6 (26.1)	
		7 (15.6)	4 (18.2)	3 (13)	
		3 (6.6)	2 (9.1)	1 (4.3)	
Suicide Intent Scale total score *N* = 3 (AD:2; DE:1)		12 (5.3)	15 (1.4)	1 (N/A)	n/a
Scale of Suicidal Ideation total *N* = 42 (AD:20; DE:22)		3.3 (5.6)	3.1 (5.3)	3.5 (5.9)	0.757^a^

* Clinical diagnosis; ^a^ Independent *t*-test; ^b^ chi square; ^c^ from Q18 on IDSC-30. Bold typeface denotes statistically significant differences.

**Table 2:** Demographic and clinical characteristics of the study population—women recruited in the perinatal period (pregnancy to 12 months following birth) and a comparison group matched for age gender and diagnosis.

Characteristics		Total *N* = 154	Perinatal *N* = 45	Non-Perinatal *N* = 109	Statistic *p*-Value
Mean Age, years (SD)		34.8 (7.1)	34.0 (6.1)	35.0 (7.3)	0·458^a^
**Marital Status**	**Single, *n* (%)** **Married/Cohabit, *n* (%)** **Other, *n* (%)**	**59 (38.3)**	**9 (20)**	**50 (45.9)**	**0·003 ^b^**
		**77 (50.0)**	**33 (73.3)**	**44 (40.4)**	
		**18 (11)**	**3 (6.7)**	**15 (13.7)**	
Clinical Diagnosis AD (%) DE (%)		78 (50.6)	22 (48.9)	56 (51.4)	0.459 ^b^
		76 (49.4)	23 (51.1)	53 (48.6)	
Diagnosis on semi-structured interview (SCAN) AD (%) DE (%)		25 (16.4)	8 (27.3)	17 (15.9)	0.472 ^b^
		127 (83.6)	37 (82.2)	90 (84.1)	
Depressive symptoms: Mean IDSC-30 total score, range 0–90 (SD)		36.6 (14.9)	37.1 (14.9)	35·8 (14·1)	0·605 ^a^
Personality: Mean SAPAS score, range 0–8 (SD)		3.2 (1.7)	3.2 (1.7)	3.2 (1.7)	0·794 ^a^
Proportion of SAPAS score ≥ 3 (%)		29 (64.4)	13 (59.1)	16 (70)	0·463 ^a^
Social Support: Mean Oslo Social Support Scale score, range 3–14 (SD)		10.3 (2.4)	10·3 (2·4)	10.0 (2·5)	0·510^a^
Life Events: Mean score on List of Threatening Events, Range 0–12 (SD)		1.5 (1.5)	1.5 (1·5)	1.9 (1.8)	0·012 ^a^
Suicidality ^c^	Nil Passive death wish Suicidal ideation Suicidal behaviour	74 (48.4)	26 (57.8)	48 (44.4)	0.159 ^b^
		30 (19.6)	9 (20)	21 (19.4)	
		23 (15)	7 (15.6)	16 (14.8)	
		26 (17)	3 (6.7)	23 (21.3)	
Suicide Intent Scale total score		12 (5.2)	12 (5.3)	12 (6.3)	1.0 ^a^
Scale of Suicidal Ideation total		3.3 (5.6)	3.3 (5.6)	4.0 (6.3)	0.581 ^a^

* Clinical diagnosis; ^a^ Independent *t*-test; ^b^ chi square; ^c^ from Q18 on IDSC-30. Bold typeface denotes statistically significant differences.
